# Polaris: Polarization of ancestral and derived polymorphic alleles for inferences of extended haplotype homozygosity in human populations

**DOI:** 10.1093/bioinformatics/btaf171

**Published:** 2025-04-12

**Authors:** Alessandro Lisi, Michael C Campbell

**Affiliations:** Department of Biological Sciences (Human and Evolutionary Biology Section), University of Southern California, Los Angeles, CA 90089, United States; Department of Biological Sciences (Human and Evolutionary Biology Section), University of Southern California, Los Angeles, CA 90089, United States

## Abstract

**Summary:**

Statistical methods that measure the extent of haplotype homozygosity on chromosomes have been highly informative for identifying episodes of recent selection. For example, the integrated haplotype score (*i*HS) and the extended haplotype homozygosity (EHH) statistics detect long-range haplotype structure around derived and ancestral alleles indicative of classic and soft selective sweeps, respectively. However, to our knowledge, there are currently no publicly available methods that classify ancestral and derived alleles in genomic datasets for the purpose of quantifying the extent of haplotype homozygosity. Here, we introduce the Polaris package, which polarizes chromosomal variants into ancestral and derived alleles and creates corresponding genetic maps for analysis by selscan and HaploSweep, two versatile haplotype-based programs that perform scans for selection. With the input files generated by Polaris, selscan and/or HaploSweep can produce the appropriate sign (either positive or negative) for outlier *i*HS statistics, enabling users to distinguish between selection on derived or ancestral alleles. In addition, Polaris can convert the numerical output of these analyses into graphical representations of selective sweeps, increasing the functionality of our software.

**Results:**

To demonstrate the utility of our approach, we applied the Polaris package to Chromosome 2 in the European Finnish, Middle Eastern Bedouin, and East African Maasai populations. More specifically, we examined the regulatory sequence in intron 13 of the *MCM6* gene associated with lactase persistence (i.e. the ability to digest the lactose sugar present in fresh milk), a region of intense interest to human evolutionary geneticists. Our analyses showed that derived alleles (at known enhancers for lactase expression) sit on an extended haplotype background in the Finnish, Bedouin, and Maasai consistent with a classic selective sweep model as determined by *i*HS and EHH statistics. Importantly, we were able to immediately identify this target allele under selection based on the information generated by our software. We also explored outlier statistics across Chromosome 2 in two distinct datasets from these populations: (i) one containing polarized alleles generated with Polaris and (ii) the other containing unpolarized alleles in the original phased vcf file. Here, we found an excess of outlier statistics on Chromosome 2 in the unpolarized datasets, raising the possibility that a subset of these “hits” of selection may be unreliable. Overall, Polaris is a versatile package that enables users to efficiently explore, interpret, and report signals of recent selection in genomic datasets.

**Availability and implementation:**

The Polaris package is free and open source on GitHub (https://github.com/alisi1989/Polaris) and DropBox (https://www.dropbox.com/scl/fo/mlxizft5267vem9u62qkn/AAnM0qX923zPzQBlPX8iteM?rlkey=uezrp4t2waffpj0nmo1evr320&e=1&st=jaodccws&dl=0).

## 1 Introduction

Positive natural selection is the process by which advantageous alleles rapidly rise to high frequency within populations, and it has played a critical role in recent human evolution (Sabeti *et al.* 2002, Liu *et al.* 2013, Zhao *et al.* 2017, Harris, Garud and DeGiorgio 2018). To date, several haplotype-based statistical tests have been developed to detect episodes of positive selection (Sabeti *et al.* 2002, 2007, Hanchard *et al.* 2006, Voight *et al.* 2006, Wang *et al.* 2006, Zhang *et al.* 2006, Cai *et al.* 2011, Han and Abney 2013, Ferrer-Admetlla *et al.* 2014, Szpiech and Hernandez 2014, Lange and Pool 2016, Zhao *et al.* 2024). Among them, the most widely used statistics are the integrated haplotype score (*i*HS; Voight *et al.* 2006) and extended haplotype homozygosity (EHH; Sabeti *et al.* 2002), which measure the length of haplotype homozygosity around adaptive alleles.

To calculate the *i*HS and EHH statistics, phased haplotypes, associated genetic maps, and in some cases the classification of polymorphisms as either ancestral or derived are typically required (depending on the software). Furthermore, the polarization of alleles can also provide a more nuanced understanding of past selective events. For example, large negative *i*HS statistics—as described in Voight *et al.* (2006)—indicate extensive haplotype homozygosity around derived alleles, which is congruent with a classic selective sweep model. Under this scenario of selection, a beneficial allele appears on a single haplotype and rises to high population frequency (Lange and Pool 2016, Booker *et al.* 2017, Rees *et al.* 2020); conversely, large positive *i*HS statistics denote long-range haplotype homozygosity around ancestral alleles indicative of selection on standing variation (an example of a soft selective sweep) (Voight *et al.* 2006). In this circumstance, ancestral alleles previously segregating neutrally become selectively advantageous in a changing environment and increase in prevalence within a population (Pritchard *et al.* 2010, Lange and Pool 2016, Rees *et al.* 2020). Given the existence of these different modes of selection, large positive and negative *i*HS statistics are of particular interest in human evolutionary studies. However, there is currently no publicly available method for categorizing ancestral and derived alleles in phased genomic datasets for the purpose of calculating these haplotype-based statistics. As a result, studies often ignore the sign (positive or negative) associated with *i*HS and primarily report the absolute values (Pickrell *et al.* 2009, Kess *et al.* 2019, Arner *et al.* 2021, Deng *et al.* 2021, Di *et al.* 2021, Klassmann and Gautier 2022, Roca-Umbert *et al.* 2022a; Randolph *et al.* 2024, Herzog *et al.* 2025). One consequence of this approach is the inability to immediately discern which allele at a given locus is adaptive as well as the possible mode of selection acting on this variation (i.e. a classic selective sweep or selection on standing variation).

To address this problem, we developed Polaris to recode phased alleles as either ancestral or derived along chromosomes and generate corresponding genetic maps for analysis by two state-of-the-art programs, selscan and HaploSweep (Szpiech and Hernandez 2014, Zhao *et al.* 2024), which scan for selection with a range of statistics, including *i*HS and EHH. These haplotype-based programs, using the input files generated by Polaris, can provide the appropriate sign (either positive or negative) to outlier statistics, enabling users to immediately distinguish between selection on derived or ancestral alleles. In addition, the Polaris package has the capability to visualize the numerical results of these analyses, facilitating the discovery, evaluation, and reporting of signals of recent selection.

## 2 Materials and methods

With the increased availability of whole genome sequencing and genomic genotyping data, researchers now have more power to identify distortions in haplotype structure following a selective sweep (Harris *et al.* 2018). While many studies of positive selection have focused on detecting classic selective sweeps (Sabeti *et al.* 2002, 2006, Voight *et al.* 2006, Enard *et al.* 2014, Uricchio *et al.* 2019), data have indicated that other forms of selection, such as soft sweeps (specifically, selection on standing or ancestral variation), have also played a key role in human adaptation (Hernandez *et al.* 2011, Messer and Petrov 2013, Schrider and Kern 2017).

**Figure 1.**
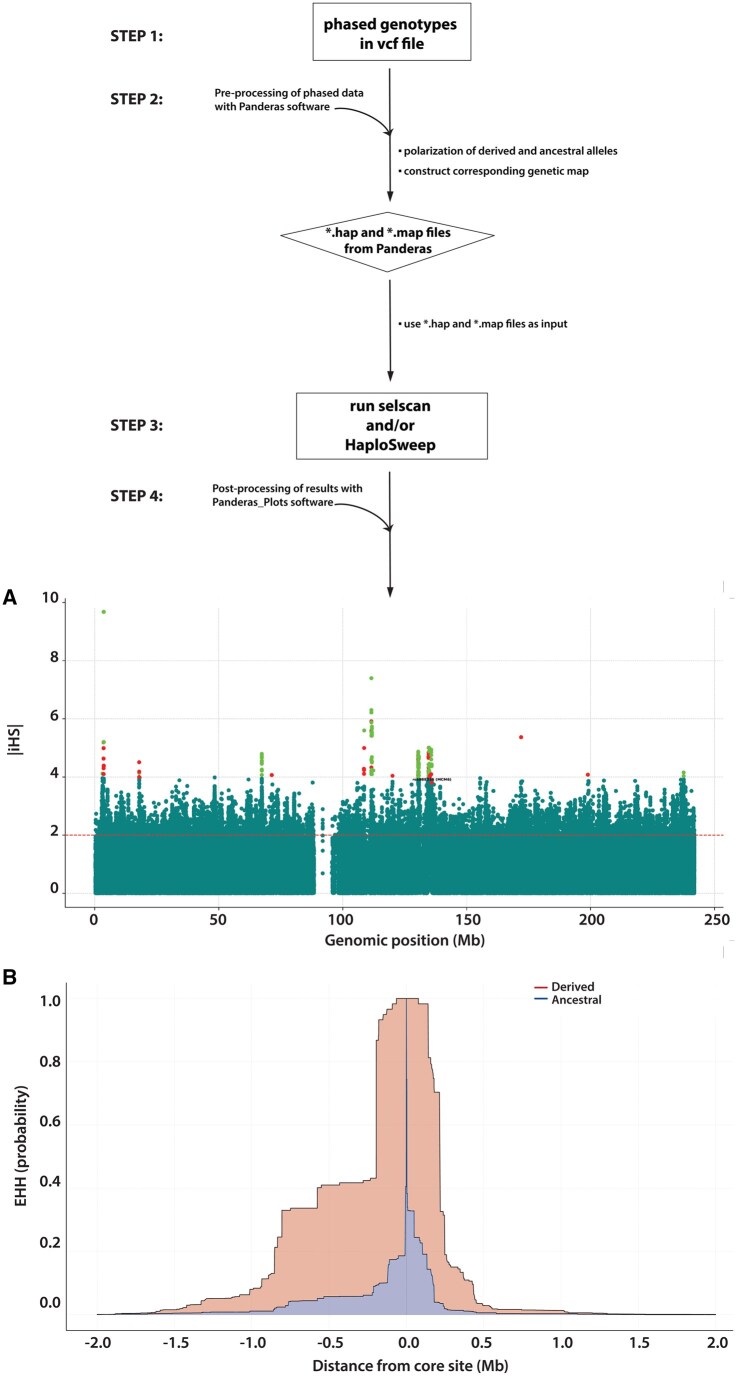
Workflow of the Polaris package. Polaris contains two distinct programs: (i) Panderas and (ii) Panderas_Plots that run in a command-line Terminal. The input for the Panderas software consists of phased bi-allelic sites in a variant call format (vcf) file without missing rs identifiers in the ID column (Step 1). The Panderas software recodes alleles in a phased vcf file as either ancestral or derived and constructs a corresponding genetic map. Then, this software generates two formatted files, *.hap and *.map, for direct use by selscan and/or HaploSweep (Steps 2 and 3). The Panderas_Plots software can visualize and annotate the tabular output of haplotype-based analyses as Manhattan and/or color-coded line plots (Step 4). For example, Panel A shows a Manhattan plot of *i*HS statistics calculated by selscan (Szpiech and Hernandez 2014) in the Finnish population from the 1000 Genomes Project. The dashed horizontal line in this plot indicates the threshold for outlier *i*HS statistics as specified by the user. We also highlighted the derived T_-13910_ allele associated with lactase persistence with a red dot and its corresponding rs identifier, rs4988235. The green and other red dots denote even more extreme *i*HS statistics. More explicitly, *i*HS > 4 (red dots) and *i*HS < −4 (green dots) denote selection on derived alleles (indicating a classic selective sweep) and selection on standing variation (indicating a soft selective sweep), respectively. Panel B shows the decay of extended haplotype homozygosity (EHH) with increasing distance from a core site (represented here by rs4988235 at distance 0.0 on the x-axis). In this EHH line graph calculated by selscan (Szpiech and Hernandez 2014), the negative and positive numbers on the *x*-axis show the distance in megabases (Mb) upstream and downstream from the core site (*rs*4988235, distance 0.0) on the forward strand, respectively; the *y*-axis is the probability that two chromosomes are homozygous at all SNPs for the interval from the core site to distance *x*. Lastly, the blue shading shows the decay of homozygosity of chromosomes carrying the ancestral allele at the core, while the red shading indicates the decay of homozygosity on chromosomes with the derived allele at the core site. The *i*HS and EHH plots for the Bedouin and Maasai can be found in the Supplementary data file online.

Polaris was developed to classify alleles as ancestral or derived along chromosomes (using existing ancestral alleles from Ensembl as a reference) and create associated genetic maps for downstream haplotype-based analyses by selscan and HaploSweep—two programs that calculate *i*HS and EHH statistics among others. After categorizing variants as either ancestral or derived, selscan and HaploSweep will provide the appropriate sign (either positive or negative) to these statistics, thereby discriminating which alleles have likely undergone a selective sweep. Polaris can also convert the tabular output of these analyses into annotated Manhattan and/or color-coded line plots, broadening its versatility (Fig. 1).

### 2.1 Polarization of ancestral and derived alleles along chromosomes

The Polaris package consists of two distinct programs written in the C-Python language: (i) Panderas and (ii) Panderas_Plots.

#### 2.1.1 Step 1: Preparation of phased vcf file

The input for Panderas should contain phased bi-allelic sites in a variant call format (vcf) file. The task of inferring haplotypes from genotype or sequence data (also known as phasing) can be accomplished with a number of publicly available programs, such as Beagle or SHAPEIT (Delaneau *et al.* 2011, Browning *et al.* 2021). Notably, all variants must have a reference SNP (rs) identifier in the ID column of the vcf file. In cases where the vcf contains missing rs identifiers (e.g. “.”), users can apply a custom Python script provided in our GitHub repository to create unique identifiers in the ID column.

#### 2.1.2 Step 2: Recode phased alleles as either ancestral or derived in a haplotype file and construct an associated genetic map

Using the phased data in the vcf file, Panderas converts “0”s and “1”s to nucleotides (e.g. A, C, G, or T) based on the allele information in the “REF” and “ALT” columns, respectively. Then, these alleles are recoded as either ancestral or derived by comparing alleles at genomic coordinates in the vcf file to ancestral alleles at the same coordinates in the Homo_sapiens_hg38_reference FASTA file from Ensembl (Cunningham *et al.* 2022; https://ftp.ensembl.org/pub/release-112/fasta/ancestral_alleles/). The Homo_sapiens_hg38_reference file, containing high-confidence calls of ancestral state, was previously generated through comparative genomics methods. More explicitly, using a multiple sequence alignment together with a phylogeny of human and nonhuman primates as input, probabilistic models were used to infer the likely ancestral sequence in humans (Paten *et al.* 2008). When a given allele at a genomic coordinate in the vcf file matches the high-confidence ancestral allele at the same coordinate in the Homo_sapiens_hg38_reference file, this allele is coded as “0” (for ancestral), and the nonancestral allele at the same coordinate in the dataset is coded as “1” (for derived). This process is applied to variation along a given chromosome, resulting in the recoding of “0”s and “1”s across individuals at each genomic coordinate. Alleles that are not coded as “0” or “1” (because there is no corresponding high-confidence ancestral allele at that position in the Homo_sapiens_hg38_reference file) are removed from the dataset.

To construct the associated genetic map, the Panderas software performs a linear interpolation to determine the genetic position (centimorgans, cM) of loci in a given dataset based on the physical position (i.e. genomic coordinate) and the corresponding genetic position in a generic PLINK map file (i.e. the reference) (Purcell *et al.* 2007, Schneider *et al.* 2017). Specifically, Panderas searches for the two physical and genetic positions in the PLINK reference file that flank a query locus in a given dataset and calculates the genetic position for that query locus using information in the reference. Next, the Panderas software generates two formatted files, *.hap (containing the recoded phased alleles) and *.map (containing chromosome number, rs identifiers, genetic positions and physical positions), that serve as input files for selscan and HaploSweep (Szpiech and Hernandez 2014, Zhao *et al.* 2024) (Fig. 1). If users wish to include a population-specific genetic map instead, they need to replace the generic PLINK map with the population-specific genetic map and allow Panderas to create the associated *.map file.

#### 2.1.3 Step 3: Run the selscan and/or HaploSweep programs using the input files generated by Panderas

The *.hap and *.map files, generated by Panderas, can be used directly as input for selscan and HaploSweep (Szpiech and Hernandez 2014, Zhao *et al.* 2024). More specific information on the usage of selscan and HaploSweep can be found at https://github.com/szpiech/selscan and https://github.com/ChenHuaLab/HaploSweep, respectively. The normalized output files from these programs will serve as input files for Panderas_Plots in Step 4.

#### 2.1.4 Step 4: Plot the numerical output of selscan and/or HaploSweep with Panderas_Plots

For the *i*HS analysis, the normalized selscan and HaploSweep output files contain standardized *i*HS statistics that have either a negative or positive sign. Extreme negative standardized values indicate extensive haplotype structure around the ancestral allele whereas extreme positive standardized values denote unusually long haplotypes surrounding the derived allele. Users should be aware that selscan and HaploSweep by convention report positive statistics when the derived allele (coded as “1”) is under selection and negative statistics when the ancestral allele (coded as “0”) is favored (Szpiech and Hernandez 2014, Roca-Umbert *et al.* 2022b; Shihabi *et al.* 2022, Szpiech 2024), which is opposite to the orientation described in Voight *et al.* (Voight *et al.* 2006).

The Panderas_Plots software also offers an array of options to visualize and annotate the tabular output of selscan and HaploSweep (Fig. 1). For example, Panderas_Plots can generate Manhattan plots of standardized statistics and insert a user-specified dashed horizontal line to indicate extreme values (standardized statistics above this threshold line will be considered outliers; Fig. 1A). Other noteworthy features include: (i) annotating specific *i*HS values with their corresponding rs identifiers using the --snps-to-highlight flag; (ii) changing the size and/or color of each dot in Manhattan plots with the --size-dots flag; and (iii) creating a list of genes harboring outlier *i*HS values in a *.txt file using --gene-annot and --gene-file. A more complete list of features can be viewed using the “--help” flag as described in the README file in our GitHub (https://github.com/alisi1989/Polaris) and DropBox (https://www.dropbox.com/scl/fo/mlxizft5267vem9u62qkn/AAnM0qX923zPzQBlPX8iteM?rlkey=uezrp4t2waffpj0nmo1evr320&e=1&st=jaodccws&dl=0) repositories. Users also have the option to save the Manhattan plots as *.pdf, *.eps, *.svg, and/or *.png files.

Furthermore, Panderas_Plots can create EHH line graphs showing the decay of haplotype homozygosity on chromosomes around ancestral and derived alleles at the same core site (Fig. 1B). In addition, Panderas_Plots automatically distinguishes these distinct chromosomes by color (specifically, red color for chromosomes with the derived allele and blue color for chromosomes carrying the ancestral allele) and provides a corresponding legend (Fig. 1B). Like the Manhattan plots, the EHH colored graphs can be saved as *.pdf, *.eps, *.svg, and/or *.png files.

## 3 Results and discussion

To our knowledge, there are currently no publicly available methods that (i) polarize alleles in phased data; (ii) create associated genetic maps for selscan and/or HaploSweep analysis; and (iii) plot the quantitative output. To demonstrate the utility of our approach, we applied the Panderas software to allelic variation on Chromosome 2 in the European Finnish, Middle Eastern Bedouin, and East African Maasai populations from the 1000 Genomes Project, the Human Genome Diversity Project (HGDP), and the haplotype map (HapMap) Project Phase 3, respectively (International HapMap Consortium 2003, Abecasis *et al.* 2012, Bergström *et al.* 2020). More explicitly, alleles along the entire length of the chromosome were recoded as either ancestral or derived in these populations for downstream analysis by selscan and HaploSweep. Next, we used the Panderas_Plots software to visualize the numerical output of these analyses. We first focused our attention on the well-studied regulatory region in intron 13 of *MCM6*, which contains polymorphisms associated with lactase persistence (Enattah *et al.* 2002, Ingram *et al.* 2007, 2009, Tishkoff *et al.* 2007, Ranciaro *et al.* 2014, Hassan *et al.* 2016, Anguita-Ruiz *et al.* 2020, Campbell and Ranciaro 2021, Lisi and Campbell 2024) and then examined other signals of selection across Chromosome 2. Our selscan and HaploSweep analyses both uncovered long-range haplotype homozygosity around the functional derived T_-13910_ allele in the Finnish (rs4988235; *i*HS > 3.5), the derived G_-__13915_ allele in the Bedouin (rs41380347; *i*HS > 3), and the derived C_-14010_ allele in the Maasai (rs145946881; *i*HS > 4), indicative of a classic selective sweep (Fig. 1; Supplementary Table S1; Supplementary Figs S1–S7). Prior studies also have reported similar genetic patterns in these populations (Enattah *et al.* 2002, Poulter *et al.* 2003, Mulcare *et al.* 2004, Tishkoff *et al.* 2007, Cramp *et al.* 2014, Ranciaro *et al.* 2014, Campbell and Ranciaro 2021). Notably, we were able to immediately identify the target allele under selection based on the sign of the *i*HS statistic (Supplementary Table S1).

Furthermore, we examined both negative and positive outlier *i*HS statistics (*i*HS< −2 and *i*HS >2, representing the most extreme 5% of empirical values) on Chromosome 2 in the Finnish, Bedouin, and Maasai (Supplementary Tables S2–S13). More specifically, we compared the number of extreme negative and positive *i*HS statistics in two separate datasets: (i) one consisting of polarized alleles generated with Panderas and (ii) one containing unpolarized alleles in the original phased vcf file on Chromosome 2. We also compared the number of extreme and non-extreme *i*HS statistics in these polarized and unpolarized datasets. Interestingly, these analyses revealed an excess of extreme negative and positive *i*HS statistics (in some cases a significant excess) in the unpolarized datasets (Supplementary Tables S2–S13). Because our software classifies alleles based on high-confidence ancestral calls in the Homo_sapiens_hg38_reference file, our polarized datasets are curated to include carefully characterized alleles. However, the loss of alleles due to this curation process (resulting in the polarized datasets) was only ∼8.5% of the total number of alleles on Chromosome 2 (Supplementary Table S14) and thus cannot completely account for the excess of outlier statistics in the unpolarized datasets. Taken together, these findings arguably suggest the possibility that a subset of the “hits” of selection on Chromosome 2 in the unpolarized datasets may be unreliable.

Moreover, of the negative outlier *i*HS statistics (*i*HS< −2) in the unpolarized datasets from the Finnish, Bedouin, and Maasai populations (which indicate selection on standing variation), both ancestral and derived alleles were coded as “0”; likewise, a mixture of ancestral and derived alleles were coded as “1” in the same datasets (Supplementary Tables S15–S20). We also observed a similar pattern for the positive outlier *i*HS statistics (*i*HS >2) in the unpolarized datasets from our populations (Supplementary Tables S15–S20). Therefore, the positive or negative sign associated with these statistics is not always informative for identifying the mode of selection (i.e. either a classic or soft selective sweep, respectively) after applying the haplotype-based methods to unpolarized alleles.

In summary, the Polaris package has the capacity to efficiently classify alleles as either ancestral or derived along chromosomes and generate associated genetic maps. Our software also provides an array of options for visualizing signals of selective sweeps. While these strengths make Polaris a powerful tool for investigating, interpreting, and summarizing patterns of selection, it is also important to note the limitations of our approach. Because we polarize alleles in a given dataset against high–confidence calls of ancestral state, alleles that match to a low-confidence ancestral allele in the dataset are subsequently removed. Although the loss of alleles will be low for many chromosomes, there are some chromosomes (e.g. 19, 21, and 22) that have a higher proportion of low-confidence ancestral alleles (∼25% to 43%; Supplementary Table S14), leading to the exclusion of the corresponding alleles in the *.hap file. While the proportions of low-confidence ancestral alleles on these chromosomes seem large, the successfully polarized alleles that remain in the *.hap file can be considered highly reliable. In addition, although strongly negative *i*HS statistics could be indicative of selection on ancestral variation in a polarized dataset, it is possible that ancestral alleles in close proximity to a derived allele under selection could exhibit large negative values due to genetic hitchhiking and may not be the direct target of selection. Therefore, as with any analysis, it is important to corroborate signals of selection from these haplotype-based methods with additional lines of evidence. Nonetheless, if prudently applied, the Polaris package is highly beneficial for investigating the nature of selection and its role in human adaptation, providing new insights into the recent evolutionary history of contemporary populations.

## Supplementary Material

btaf171_Supplementary_Data

## Data Availability

The data underlying this article are available in the 1000 Genomes Consortium Project at https://www.internationalgenome.org/data-portal/population/FIN; in the HGDP at https://ngs.sanger.ac.uk/production/hgdp/hgdp_wgs.20190516/statphase/; and in the HapMap Project Phase 3 at https://ftp.ncbi.nlm.nih.gov/hapmap/phase_3/. To analyze the LP-associated allele and increase the density of variants in the Maasai from the HapMap Project, we imputed genotypes on Chromosome 2 in this population using the TOPMed Imputation Server (version 3 reference panel) and retained imputed sites with *r*^2^ ≥ 0.9. The reference overlap with the Maasai was 97.68%.

## References

[btaf171-B1] Abecasis GR , Auton A, Brooks LD et al; 1000 Genomes Project Consortium. An integrated map of genetic variation from 1,092 human genomes. Nature 2012;491:56–65. 23128226 10.1038/nature11632 PMC3498066

[btaf171-B2] Anguita-Ruiz A , Aguilera CM, Gil Á. Genetics of lactose intolerance: an updated review and online interactive world maps of phenotype and genotype frequencies. Nutrients 2020;12:2689. 32899182 10.3390/nu12092689 PMC7551416

[btaf171-B3] Arner AM , Grogan KE, Grabowski M et al Patterns of recent natural selection on genetic loci associated with sexually differentiated human body size and shape phenotypes. PLoS Genet 2021;17:e1009562. 34081690 10.1371/journal.pgen.1009562 PMC8174730

[btaf171-B4] Bergström A , McCarthy SA, Hui R et al Insights into human genetic variation and population history from 929 diverse genomes. Science 2020;367:eaay5012. 32193295 10.1126/science.aay5012 PMC7115999

[btaf171-B5] Booker TR , Jackson BC, Keightley PD. Detecting positive selection in the genome. BMC Biol 2017;15:98. 29084517 10.1186/s12915-017-0434-y PMC5662103

[btaf171-B6] Browning BL , Tian X, Zhou Y et al Fast two-stage phasing of large-scale sequence data. Am J Hum Genet 2021;108:1880–90. 34478634 10.1016/j.ajhg.2021.08.005 PMC8551421

[btaf171-B7] Cai Z , Camp NJ, Cannon-Albright L et al Identification of regions of positive selection using shared genomic segment analysis. Eur J Hum Genet 2011;19:667–71. 21304558 10.1038/ejhg.2010.257 PMC3110045

[btaf171-B8] Campbell MC , Ranciaro A. Human adaptation, demography and cattle domestication: an overview of the complexity of lactase persistence in Africa. Hum Mol Genet 2021; 30: R98–109. 33847744 10.1093/hmg/ddab027

[btaf171-B9] Cramp LJE , Evershed RP, Lavento M et al Neolithic dairy farming at the extreme of agriculture in Northern Europe. Proc Biol Sci 2014;281:20140819. 25080345 10.1098/rspb.2014.0819 PMC4132672

[btaf171-B10] Cunningham F , Allen JE, Allen J et al Ensembl 2022. Nucleic Acids Res 2022; 50:D988–95. 34791404 10.1093/nar/gkab1049 PMC8728283

[btaf171-B11] Delaneau O , Marchini J, Zagury J-F. A linear complexity phasing method for thousands of genomes. Nat Methods 2011;9:179–81. 22138821 10.1038/nmeth.1785

[btaf171-B12] Deng Z , Zhen J, Harrison GF et al Adaptive admixture of HLA class I allotypes enhanced genetically determined strength of natural killer cells in East Asians. Mol Biol Evol 2021;38:2582–96. 33616658 10.1093/molbev/msab053 PMC8136484

[btaf171-B13] Di C , Murga Moreno J, Salazar-Tortosa DF et al Decreased recent adaptation at human mendelian disease genes as a possible consequence of interference between advantageous and deleterious variants. Elife 2021;10:e69026. 10.7554/eLife.69026 PMC8526059 34636724

[btaf171-B14] Enard D , Messer PW, Petrov DA. Genome-wide signals of positive selection in human evolution. Genome Res 2014;24:885–95. 24619126 10.1101/gr.164822.113 PMC4032853

[btaf171-B15] Enattah NS , Sahi T, Savilahti E et al Identification of a variant associated with adult-type hypolactasia. Nat Genet 2002;30:233–7. 11788828 10.1038/ng826

[btaf171-B16] Ferrer-Admetlla A , Liang M, Korneliussen T et al On detecting incomplete soft or hard selective sweeps using haplotype structure. Mol Biol Evol 2014;31:1275–91. 24554778 10.1093/molbev/msu077 PMC3995338

[btaf171-B17] Han L , Abney M. Using identity by descent estimation with dense genotype data to detect positive selection. Eur J Hum Genet 2013;21:205–11. 22781100 10.1038/ejhg.2012.148 PMC3548265

[btaf171-B18] Hanchard NA , Rockett KA, Spencer C et al Screening for recently selected alleles by analysis of human haplotype similarity. Am J Hum Genet 2006;78:153–9. 16385459 10.1086/499252 PMC1380214

[btaf171-B19] Harris AM , Garud NR, DeGiorgio M. Detection and classification of hard and soft sweeps from unphased genotypes by multilocus genotype identity. Genetics 2018;210:1429–52. 30315068 10.1534/genetics.118.301502 PMC6283157

[btaf171-B20] Hassan HY , van Erp A, Jaeger M et al Genetic diversity of lactase persistence in East African populations. BMC Res Notes 2016;9:8. 26728963 10.1186/s13104-015-1833-1 PMC4700599

[btaf171-B21] Hernandez RD , Kelley JL, Elyashiv E, 1000 Genomes Project et al Classic selective sweeps were rare in recent human evolution. Science 2011;331:920–4. 21330547 10.1126/science.1198878 PMC3669691

[btaf171-B22] Herzog T , Larena M, Kutanan W et al Natural selection and adaptive traits in the Maniq, a nomadic hunter-gatherer society from Mainland Southeast Asia. Sci Rep 2025;15:4809. 39924514 10.1038/s41598-024-83657-0 PMC11808089

[btaf171-B23] Ingram CJE , Elamin MF, Mulcare CA et al A novel polymorphism associated with lactose tolerance in Africa: multiple causes for lactase persistence? Hum Genet 2007;120:779–88. 17120047 10.1007/s00439-006-0291-1

[btaf171-B24] Ingram CJE , Mulcare CA, Itan Y et al Lactose digestion and the evolutionary genetics of lactase persistence. Hum Genet 2009;124:579–91. 19034520 10.1007/s00439-008-0593-6

[btaf171-B25] International HapMap Consortium. The international HapMap project. Nature 2003;426:789–96. 14685227 10.1038/nature02168

[btaf171-B26] Kess T , Bentzen P, Lehnert SJ et al A migration-associated supergene reveals loss of biocomplexity in Atlantic cod. Sci Adv 2019;5:eaav2461. 31249864 10.1126/sciadv.aav2461 PMC6594766

[btaf171-B27] Klassmann A , Gautier M. Detecting selection using extended haplotype homozygosity (EHH)-based statistics in unphased or unpolarized data. PLoS One 2022; 17: e0262024. 35041674 10.1371/journal.pone.0262024 PMC8765611

[btaf171-B28] Lange JD , Pool JE. A haplotype method detects diverse scenarios of local adaptation from genomic sequence variation. Mol Ecol 2016;25:3081–100. 27135633 10.1111/mec.13671 PMC4931985

[btaf171-B29] Lisi A , Campbell MC. AncestryGrapher toolkit: Python command-line pipelines to visualize global- and local-ancestry inferences from the RFMIX version 2 software. Bioinformatics 2024;40:btae616. 10.1093/bioinformatics/btae616 PMC11534077 39412440

[btaf171-B30] Liu X , Ong RT-H, Pillai EN et al Detecting and characterizing genomic signatures of positive selection in global populations. Am J Hum Genet 2013;92:866–81. 23731540 10.1016/j.ajhg.2013.04.021 PMC3675259

[btaf171-B31] Messer PW , Petrov DA. Population genomics of rapid adaptation by soft selective sweeps. Trends Ecol Evol 2013;28:659–69. 24075201 10.1016/j.tree.2013.08.003 PMC3834262

[btaf171-B32] Mulcare CA , Weale ME, Jones AL et al The T allele of a single-nucleotide polymorphism 13.9 kb upstream of the lactase gene (LCT) (C-13.9kbT) does not predict or cause the lactase-persistence phenotype in Africans. Am J Hum Genet 2004;74:1102–10. 15106124 10.1086/421050 PMC1182074

[btaf171-B33] Paten B , Herrero J, Fitzgerald S et al Genome-wide nucleotide-level mammalian ancestor reconstruction. Genome Res 2008;18:1829–43. 18849525 10.1101/gr.076521.108 PMC2577868

[btaf171-B34] Pickrell JK , Coop G, Novembre J et al Signals of recent positive selection in a worldwide sample of human populations. Genome Res 2009;19:826–37. 19307593 10.1101/gr.087577.108 PMC2675971

[btaf171-B35] Poulter M , Hollox E, Harvey CB et al The causal element for the lactase persistence/non-persistence polymorphism is located in a 1 Mb region of linkage disequilibrium in Europeans. Ann Hum Genet 2003;67:298–311. 12914565 10.1046/j.1469-1809.2003.00048.x

[btaf171-B36] Pritchard JK , Pickrell JK, Coop G. The genetics of human adaptation: hard sweeps, soft sweeps, and polygenic adaptation. Curr Biol 2010;20:R208–15. 20178769 10.1016/j.cub.2009.11.055 PMC2994553

[btaf171-B37] Purcell S , Neale B, Todd-Brown K et al PLINK: a tool set for whole-genome association and population-based linkage analyses. Am J Hum Genet 2007;81:559–75. 17701901 10.1086/519795 PMC1950838

[btaf171-B38] Ranciaro A , Campbell MC, Hirbo JB et al Genetic origins of lactase persistence and the spread of pastoralism in Africa. Am J Hum Genet 2014;94:496–510. 24630847 10.1016/j.ajhg.2014.02.009 PMC3980415

[btaf171-B39] Randolph HE , Aracena KA, Lin Y-L et al Shaping immunity: the influence of natural selection on population immune diversity. Immunol Rev 2024;323:227–40. 38577999 10.1111/imr.13329

[btaf171-B40] Rees JS , Castellano S, Andrés AM. The genomics of human local adaptation. Trends Genet 2020;36:415–28. 32396835 10.1016/j.tig.2020.03.006

[btaf171-B41] Roca-Umbert A , Caro-Consuegra R, Londono-Correa D et al Understanding signatures of positive natural selection in human zinc transporter genes. Sci Rep 2022a;12:4320. 35279701 10.1038/s41598-022-08439-y PMC8918337

[btaf171-B42] Roca-Umbert A , Caro-Consuegra R, Londono-Correa D et al Author correction: understanding signatures of positive natural selection in human zinc transporter genes. Sci Rep 2022b;12:5378. 35354914 10.1038/s41598-022-09566-2 PMC8967874

[btaf171-B43] Sabeti PC , Reich DE, Higgins JM et al Detecting recent positive selection in the human genome from haplotype structure. Nature 2002;419:832–7. 12397357 10.1038/nature01140

[btaf171-B44] Sabeti PC , Schaffner SF, Fry B et al Positive natural selection in the human lineage. Science 2006;312:1614–20. 16778047 10.1126/science.1124309

[btaf171-B45] Sabeti PC , Varilly P, Fry B et al; International HapMap Consortium. Genome-wide detection and characterization of positive selection in human populations. Nature 2007;449:913–8. 17943131 10.1038/nature06250 PMC2687721

[btaf171-B46] Schrider DR , Kern AD. Soft sweeps are the dominant mode of adaptation in the human genome. Mol Biol Evol 2017;34:1863–77. 28482049 10.1093/molbev/msx154 PMC5850737

[btaf171-B47] Schneider VA , Graves-Lindsay T, Howe K et al Evaluation of GRCh38 and de novo haploid genome assemblies demonstrates the enduring quality of the reference assembly. Genome Res 2017;27:849–64. 28396521 10.1101/gr.213611.116 PMC5411779

[btaf171-B48] Shihabi M , Lukic B, Cubric-Curik V et al Identification of selection signals on the X-chromosome in East Adriatic sheep: a new complementary approach. Front Genet 2022;13:887582. 35615375 10.3389/fgene.2022.887582 PMC9126029

[btaf171-B49] Szpiech ZA. Selscan 2.0: scanning for sweeps in unphased data. Bioinformatics 2024;40:btae006. 10.1093/bioinformatics/btae006 PMC10789311 38180866

[btaf171-B50] Szpiech ZA , Hernandez RD. Selscan: an efficient multi-threaded program to perform EHH-based scans for positive selection. *Mol Biol Evol* 2014;31:2824–7. 10.1093/molbev/msu211 PMC4166924 25015648

[btaf171-B51] Tishkoff SA , Reed FA, Ranciaro A et al Convergent adaptation of human lactase persistence in Africa and Europe. Nat Genet 2007;39:31–40. 17159977 10.1038/ng1946 PMC2672153

[btaf171-B52] Uricchio LH , Kitano HC, Gusev A et al An evolutionary compass for detecting signals of polygenic selection and mutational bias. Evol Lett 2019;3:69–79. 30788143 10.1002/evl3.97 PMC6369964

[btaf171-B53] Voight BF , Kudaravalli S, Wen X et al A map of recent positive selection in the human genome. PLoS Biol 2006;4:e72. 16494531 10.1371/journal.pbio.0040072 PMC1382018

[btaf171-B54] Wang ET , Kodama G, Baldi P et al Global landscape of recent inferred Darwinian selection for Homo sapiens. Proc Natl Acad Sci USA 2006;103:135–40. 16371466 10.1073/pnas.0509691102 PMC1317879

[btaf171-B55] Zhang C , Bailey DK, Awad T et al A whole genome long-range haplotype (WGLRH) test for detecting imprints of positive selection in human populations. Bioinformatics 2006;22:2122–8. 16845142 10.1093/bioinformatics/btl365

[btaf171-B56] Zhao S , Chi L, Fu M et al HaploSweep: detecting and distinguishing recent soft and hard selective sweeps through haplotype structure. Mol Biol Evol 2024;41:msae192. 10.1093/molbev/msae192 PMC11452351 39288167

[btaf171-B57] Zhao Z-M , Campbell MC, Li N et al Detection of regional variation in selection intensity within protein-coding genes using DNA sequence polymorphism and divergence. Mol Biol Evol 2017;34:3006–22. 28962009 10.1093/molbev/msx213 PMC5850860

