# Supplementary material for: Polaris: Polarization of ancestral and derived polymorphic alleles for inferences of extended haplotype homozygosity in human populations
Source: Bioinformatics. 2025 Apr 12;41(6):btaf171. doi: 10.1093/bioinformatics/btaf171 (PMC12199141; doi:10.1093/bioinformatics/btaf171)
Supplement: btaf171_Supplementary_Data [file btaf171_supplementary_data.pdf]

Supplementary Tables

Table S1—Truncated output from selscan and HaploSweep analyses. This Table shows the truncated normalized output of the selscan and HaploSweep, focusing on LP-associated single nucleotide polymorphisms (SNPs) in intron 13 of the *MCM6* gene. For each SNP (rs4988235, rs41380347, and rs145946881), we provide the locus ID, chromosome number, physical position (genomic coordinate), derived allele frequency, the *i*HS statistic, and the method used to calculate the statistic.

Table S2— Distribution of extreme positive and negative *i*HS statistics (*i*HS < -2 and *i*HS > 2) on Chromosome 2 in the Finnish population from the 1000 Genomes Project calculated by selscan. We compared the number of extreme negative and positive *i*HS statistics—calculated by selscan (Szpiech and Hernandez 2014) —in two distinct datasets: i) one containing polarized alleles generated with Panderas and ii) one containing unpolarized alleles in the original phased vcf file on Chromosome 2 in a 2 × 2 contingency table. The chi-square statistic (χ^2) statistic was used to measure the divergence between observed and expected data. Statistical significance was assessed at $P < 0.05$, which represents the probability of the observed data occurring by chance alone.

Table S3— Distribution of extreme positive and negative *i*HS statistics (*i*HS < -2 and *i*HS > 2) on Chromosome 2 in the Bedouin population from the HGDP Project calculated by selscan. We compared the number of extreme negative and positive *i*HS statistics—calculated by selscan (Szpiech and Hernandez 2014) —in two distinct datasets: i) one containing polarized alleles generated with Panderas and ii) one containing unpolarized alleles in the original phased vcf file on Chromosome 2 in a 2 × 2 contingency table. The chi-square statistic (χ^2) statistic was used to measure the divergence between observed and expected data. Statistical significance was assessed at $P < 0.05$, which represents the probability of the observed data occurring by chance alone.

Table S4— Distribution of extreme positive and negative *i*HS statistics (*i*HS < -2 and *i*HS > 2) on Chromosome 2 in the Maasai population from the HapMap Project calculated by selscan. We compared the number of extreme negative and positive *i*HS statistics—calculated by selscan (Szpiech and Hernandez 2014) —in two distinct datasets: i) one containing polarized alleles generated with Panderas and ii) one containing unpolarized alleles in the original phased vcf file on Chromosome 2 in a 2 × 2 contingency table. The chi-square statistic (χ^2) statistic was used to measure the divergence between observed and expected data. Statistical significance was assessed at $P < 0.05$, which represents the probability of the observed data occurring by chance alone.

Table S5—Distribution of extreme positive and negative *i*HS statistics (*i*HS < -2 and *i*HS > 2) on Chromosome 2 in the Finnish population from the 1000 Genomes Project calculated by HaploSweep. We compared the number of extreme negative and positive *i*HS statistics—calculated by HaploSweep (Zhao et al. 2024) —in two separate datasets: i) one containing polarized alleles (generated with Panderas) and ii) one containing

unpolarized alleles in the original phased vcf file on Chromosome 2 in a 2 × 2 contingency table. The chi-square statistic (χ^2) statistic was used to measure the divergence between observed and expected data. Statistical significance was assessed at $P < 0.05$.

Table S6— Distribution of extreme positive and negative *i*HS statistics (*i*HS < -2 and *i*HS > 2) on Chromosome 2 in the Bedouin population from the HGDP Project calculated by HaploSweep. We compared the number of extreme negative and positive *i*HS statistics—calculated by HaploSweep (Zhao et al. 2024) —in two separate datasets: i) one containing polarized alleles (generated with Panderas) and ii) one containing unpolarized alleles in the original phased vcf file on Chromosome 2 in a 2 × 2 contingency table. The chi-square statistic (χ^2) statistic was used to measure the divergence between observed and expected data. Statistical significance was assessed at $P < 0.05$.

Table S7—Distribution of extreme positive and negative *i*HS statistics (*i*HS < -2 and *i*HS > 2) on Chromosome 2 in the Maasai population from the HapMap Project calculated by HaploSweep. We compared the number of extreme negative and positive *i*HS statistics—calculated by HaploSweep (Zhao et al. 2024) —in two separate datasets: i) one containing polarized alleles (generated with Panderas) and ii) one containing unpolarized alleles in the original phased vcf file on Chromosome 2 in a 2 × 2 contingency table. The chi-square statistic (χ^2) statistic was used to measure the divergence between observed and expected data. Statistical significance was assessed at $P < 0.05$.

Table S8— Distribution of extreme *i*HS statistics and non-extreme *i*HS statistics on Chromosome 2 in the Finnish population from the 1000 Genomes Project calculated by selscan. Here, we compared the number of extreme *i*HS statistics (defined as *i*HS < -2 and *i*HS > 2) and non-extreme *i*HS statistics (defined as *i*HS ≥ -2 and *i*HS ≤ 2)—calculated by selscan (Szpiech and Hernandez 2014)—in two separate datasets: i) one containing polarized alleles (generated with Panderas) and ii) one containing unpolarized alleles in the original phased vcf file on Chromosome 2 in a 2 × 2 contingency table. The chi-square statistic (χ^2) statistic was used to measure the divergence between observed and expected data. Statistical significance was assessed at $P < 0.05$.

Table S9— Distribution of extreme *i*HS statistics and non-extreme *i*HS statistics on Chromosome 2 in the Bedouin population from the HGDP Panel calculated by selscan. Here, we compared the number of extreme *i*HS statistics (defined as *i*HS < -2 and *i*HS > 2) and non-extreme *i*HS statistics (defined as *i*HS ≥ -2 and *i*HS ≤ 2)—calculated by selscan (Szpiech and Hernandez 2014)—in two separate datasets: i) one containing polarized alleles (generated with Panderas) and ii) one containing unpolarized alleles in the original phased vcf file on Chromosome 2 in a 2 × 2 contingency table. The chi-square statistic (χ^2) statistic was used to measure the divergence between observed and expected data. Statistical significance was assessed at $P < 0.05$.

Table S10— Distribution of extreme *i*HS statistics and non-extreme *i*HS statistics on Chromosome 2 in the Maasai population from the HapMap Project calculated by selscan. Here, we compared the number of extreme *i*HS statistics (defined as *i*HS < -2 and *i*HS > 2) and non-extreme *i*HS statistics (defined as *i*HS ≥ -2 and *i*HS ≤ 2**)**—calculated by selscan (Szpiech and Hernandez 2014)—in two separate datasets: i) one containing polarized alleles (generated with Panderas) and ii) one containing unpolarized alleles in the original phased vcf file on Chromosome 2 in a 2 × 2 contingency table. The chi-square statistic (χ^2) statistic was used to measure the divergence between observed and expected data. Statistical significance was assessed at $P < 0.05$.

Table S11— Distribution of extreme *i*HS statistics and non-extreme *i*HS statistics on Chromosome 2 in the Finnish population from the 1000 Genomes Project calculated by HaploSweep. Here, we compared the number of extreme *i*HS statistics (defined as *i*HS < -2 and *i*HS > 2) and non-extreme *i*HS statistics (defined as *i*HS ≥ -2 and *i*HS ≤ 2**)**—calculated by HaploSweep (Zhao et al. 2024)—in two separate datasets: i) one containing polarized alleles (generated with Panderas) and ii) one containing unpolarized alleles in the original phased vcf file on Chromosome 2 in a 2 × 2 contingency table. The chi-square statistic (χ^2) statistic was used to measure the divergence between observed and expected data. Statistical significance was assessed at $P < 0.05$.

Table S12— Distribution of extreme *i*HS statistics and non-extreme *i*HS statistics on Chromosome 2 in the Bedouin population from the HGDP Panel calculated by HaploSweep. Here, we compared the number of extreme *i*HS statistics (defined as *i*HS < -2 and *i*HS > 2) and non-extreme *i*HS statistics (defined as *i*HS ≥ -2 and *i*HS ≤ 2**)**—calculated by HaploSweep (Zhao et al. 2024)—in two separate datasets: i) one containing polarized alleles (generated with Panderas) and ii) one containing unpolarized alleles in the original phased vcf file on Chromosome 2 in a 2 × 2 contingency table. The chi-square statistic (χ^2) statistic was used to measure the divergence between observed and expected data. Statistical significance was assessed at $P < 0.05$.

Table S13— Distribution of extreme *i*HS statistics and non-extreme *i*HS statistics on Chromosome 2 in the Maasai population from the HapMap Project calculated by HaploSweep. Here, we compared the number of extreme *i*HS statistics (defined as *i*HS < -2 and *i*HS > 2) and non-extreme *i*HS statistics (defined as *i*HS ≥ -2 and *i*HS ≤ 2**)**—calculated by HaploSweep (Zhao et al. 2024)—in two separate datasets: i) one containing polarized alleles (generated with Panderas) and ii) one containing unpolarized alleles in the original phased vcf file on Chromosome 2 in a 2 × 2 contingency table. The chi-square statistic (χ^2) statistic was used to measure the divergence between observed and expected data. Statistical significance was assessed at $P < 0.05$.

Table S14— Proportion of nucleotide sites that are not classified as either ancestral or derived across the autosomal genome. This Table shows the total number of high-confidence and low-confidence ancestral alleles per autosomal chromosome (this count does not include gaps, which indicate the absence of a particular allele in humans) in the

Homo_sapiens_hg38_reference file. Furthermore, we show the number of low-confidence ancestral alleles (not including gaps) and the proportion of low-confidence ancestral alleles per chromosome. Importantly, low-confidence alleles are not used to categorize alleles as ancestral or derived in a given dataset.

Table S15— Summary statistics for unpolarized SNPs on Chromosome 2 in the Finnish population from the 1000 Genomes Project (selscan). Here, we examined both negative and positive outlier *i*HS statistics (*i*HS < -2 and *i*HS > 2) calculated by selscan (Szpiech and Hernandez 2014), representing the most extreme 5% of empirical values on Chromosome 2. Of the negative outlier statistics indicating selection on standing variation in the unpolarized dataset, the alleles coded as ‘0’ are a mix of ancestral and derived alleles (as opposed to one allele state only). Similarly, of the positive outlier *i*HS statistics indicating a classic selective sweep, the alleles coded as ‘1’ in the same dataset are a mixture of ancestral and derived alleles. The proportion of ancestral alleles coded as ‘1’ is ~10%, while the proportion of derived alleles coded as ‘0’ is ~70%.

Table S16— Summary statistics for unpolarized SNPs on Chromosome 2 in the Bedouin population from the HGDP Project (selscan). Here, we examined both negative and positive outlier *i*HS statistics (*i*HS < -2 and *i*HS > 2) calculated by selscan (Szpiech and Hernandez 2014), representing the most extreme 5% of empirical values on Chromosome 2. Of the negative outlier statistics indicating selection on standing variation in the unpolarized dataset, the alleles coded as ‘0’ are a mix of ancestral and derived alleles (as opposed to one allele state only). Similarly, of the positive outlier *i*HS statistics indicating a classic selective sweep, the alleles coded as ‘1’ in the same dataset are a mixture of ancestral and derived alleles. The proportion of ancestral alleles coded as ‘1’ is ~40%, while the proportion of derived alleles coded as ‘0’ is ~26%.

Table S17— Summary statistics for unpolarized SNPs on Chromosome 2 in the Maasai population from the HapMap Project (selscan). Here, we examined both negative and positive outlier *i*HS statistics (*i*HS < -2 and *i*HS > 2) calculated by selscan (Szpiech and Hernandez 2014), representing the most extreme 5% of empirical values on Chromosome 2. Of the negative outlier statistics indicating selection on standing variation in the unpolarized dataset, the alleles coded as ‘0’ are a mix of ancestral and derived alleles (as opposed to one allele state only). Similarly, of the positive outlier *i*HS statistics indicating a classic selective sweep, the alleles coded as ‘1’ in the same dataset are a mixture of ancestral and derived alleles. The proportion of ancestral alleles coded as ‘1’ is ~30%, while the proportion of derived alleles coded as ‘0’ is ~28%.

Table S18— Summary statistics for unpolarized SNPs on Chromosome 2 in the Finnish population from the 1000 Genomes Project (HaploSweep). Here, we examined both negative and positive outlier *i*HS statistics (*i*HS < -2 and *i*HS > 2) calculated by HaploSweep (Zhao et al. 2024), representing the most extreme 5% of empirical values on Chromosome 2. Of the negative outlier statistics indicating selection on standing variation in the unpolarized dataset, the alleles coded as ‘0’ are a mix of ancestral and derived alleles (as

opposed to one allele state only). Similarly, of the positive outlier *i*HS statistics indicating a classic selective sweep, the alleles coded as ‘1’ in the same dataset are a mixture of ancestral and derived alleles. The proportion of ancestral alleles coded as ‘1’ is ~23%, while the proportion of derived alleles coded as ‘0’ is ~59%.

Table S19— Summary statistics for unpolarized SNPs on Chromosome 2 in the Bedouin population from the HDGP Project (HaploSweep). Here, we examined both negative and positive outlier *i*HS statistics (*i*HS < -2 and *i*HS > 2) calculated by HaploSweep (Zhao et al. 2024), representing the most extreme 5% of empirical values on Chromosome 2. Of the negative outlier statistics indicating selection on standing variation in the unpolarized dataset, the alleles coded as ‘0’ are a mix of ancestral and derived alleles (as opposed to one allele state only). Similarly, of the positive outlier *i*HS statistics indicating a classic selective sweep, the alleles coded as ‘1’ in the same dataset are a mixture of ancestral and derived alleles. The proportion of ancestral alleles coded as ‘1’ is ~32%, while the proportion of derived alleles coded as ‘0’ is ~32%.

Table S20— Summary statistics for unpolarized SNPs on Chromosome 2 in the Maasai population from the HapMap Project (HaploSweep). Here, we examined both negative and positive outlier *i*HS statistics (*i*HS < -2 and *i*HS > 2) calculated by HaploSweep (Zhao et al. 2024), representing the most extreme 5% of empirical values on Chromosome 2. Of the negative outlier statistics indicating selection on standing variation in the unpolarized dataset, the alleles coded as ‘0’ are a mix of ancestral and derived alleles (as opposed to one allele state only). Similarly, of the positive outlier *i*HS statistics indicating a classic selective sweep, the alleles coded as ‘1’ in the same dataset are a mixture of ancestral and derived alleles. The proportion of ancestral alleles coded as ‘1’ is ~28%, while the proportion of derived alleles coded as ‘0’ is ~31%.

Supplementary Figures

Figure S1: Plots of *i*HS and EHH statistics on Chromosome 2, calculated by selscan using polarized data, in the Bedouin population from the HGDP Project. **Panel A** shows a Manhattan plot of *i*HS statistics calculated by selscan (Szpiech and Hernandez 2014). The dashed horizontal line indicates the threshold for outlier *i*HS statistics as specified by the user (in this case *i*HS = 2; statistics above this line are considered outliers). Here, we highlighted the derived G_{-13915} allele associated with lactase persistence in the Bedouin population with a red dot and its corresponding rs identifier (rs41380347). The green and other red dots denote very extreme *i*HS statistics. More explicitly, *i*HS > 4 (red dots) and *i*HS < -4 (green dots) indicate selection on derived alleles (suggestive of a classic selective sweep) and selection on standing variation (suggestive of a soft selective sweep), respectively. **Panel B** shows the decay of EHH (haplotype homozygosity) with increasing distance from the rs41380347 core site, which is associated with lactase persistence. In this EHH line graph, the distance from the core SNP (at zero) is displayed in megabases (Mb) on the x-axis; the negative numbers indicate the distance upstream from the core SNP, while positive values indicate distance downstream from the core SNP on the forward strand. The

y-axis is the probability that two chromosomes are homozygous at all SNPs for the interval from the core site to distance x. Lastly, the blue line represents the decay of homozygosity of chromosomes carrying the ancestral allele at the core, while the red line signifies the decay of homozygosity on chromosomes carrying the derived allele at the core site.

Figure S2: Plots of *i*HS and EHH statistics on Chromosome 2, calculated by selscan using polarized data, in the Maasai population from the HapMap Project. **Panel A** shows a Manhattan plot of *i*HS statistics calculated by selscan (Szpiech and Hernandez 2014). The dashed horizontal line indicates the threshold for outlier *i*HS statistics as specified by the user (in this case *i*HS = 2; statistics above this line are considered outliers). Here, we highlighted the derived C_{-14010} allele associated with lactase persistence in the Bedouin population with a red dot and its corresponding rs identifier (rs145946881). The green and other red dots denote very extreme *i*HS statistics. More explicitly, *i*HS > 4 (red dots) and *i*HS < -4 (green dots) indicate selection on derived alleles (suggestive of a classic selective sweep) and selection on standing variation (suggestive of a soft selective sweep), respectively. **Panel B** shows the decay of EHH (haplotype homozygosity) with increasing distance from the rs145946881 core site, which is associated with lactase persistence. In this EHH line graph, the distance from the core SNP (at zero) is displayed in megabases (Mb) on the x-axis; the negative numbers indicate the distance upstream from the core SNP, while positive values indicate distance downstream from the core SNP on the forward strand. The y-axis is the probability that two chromosomes are homozygous at all SNPs for the interval from the core site to distance x. Lastly, the blue line represents the decay of homozygosity of chromosomes carrying the ancestral allele at the core, while the red line signifies the decay of homozygosity on chromosomes carrying the derived allele at the core site.

Figure S3: Plots of *i*HS and EHH statistics on Chromosome 2, calculated by HaploSweep using polarized data, in the Finnish population from the 1000 Genomes Project. **Panel A** shows a Manhattan plot of *i*HS statistics calculated by HaploSweep (Zhao et al. 2024). The dashed horizontal line indicates the threshold for outlier *i*HS statistics as specified by the user (statistics above this line are considered outliers). Here, we highlighted the derived T_{-13910} allele associated with lactase persistence in the Finnish population with a red dot and its corresponding rs identifier (rs4988235). The green and other red dots denote very extreme *i*HS statistics. More explicitly, *i*HS > 4 (red dots) and *i*HS < -4 (green dots) indicate selection on derived alleles (suggestive of a classic selective sweep) and selection on standing variation (suggestive of a soft selective sweep), respectively. **Panel B** shows the decay of EHH (haplotype homozygosity) with increasing distance from the rs4988235 core site, which is associated with lactase persistence. In this EHH line graph, the x-axis shows physical position distance on Chromosome 2 in megabases (Mb), and the y-axis is the probability that two chromosomes are homozygous at all SNPs for the interval from the core site to distance x. Lastly, the blue line represents the decay of homozygosity of chromosomes carrying the ancestral allele at the core, while the red line signifies the decay of homozygosity on chromosomes carrying the derived allele at the core site.

Figure S4: Plots of *i*HS and EHH statistics on Chromosome 2, calculated by HaploSweep using polarized data, in the Bedouin population from the HGDP Project. **Panel A** shows a Manhattan plot of *i*HS statistics calculated by HaploSweep (Zhao et al. 2024). The dashed horizontal line indicates the threshold for outlier *i*HS statistics as specified by the user (in this case *i*HS = 2; statistics above this line are considered outliers). Here, we highlighted the derived G_{-13915} allele associated with lactase persistence in the Bedouin population with a red dot and its corresponding rs identifier (rs41380347). The green and other red dots denote very extreme *i*HS statistics. More explicitly, *i*HS > 4 (red dots) and *i*HS < -4 (green dots) indicate selection on derived alleles (suggestive of a classic selective sweep) and selection on standing variation (suggestive of a soft selective sweep), respectively. **Panel B** shows the decay of EHH (haplotype homozygosity) with increasing distance from the rs41380347 core site, which is associated with lactase persistence. In this EHH line graph, the x-axis shows physical position distance on Chromosome 2 in megabases (Mb), and the y-axis is the probability that two chromosomes are homozygous at all SNPs for the interval from the core site to distance x. Lastly, the blue line represents the decay of homozygosity of chromosomes carrying the ancestral allele at the core, while the red line signifies the decay of homozygosity on chromosomes carrying the derived allele at the core site.

Figure S5: Plots of *i*HS and EHH statistics on Chromosome 2, calculated by HaploSweep using polarized data, in the Maasai population from the HapMap Project. **Panel A** shows a Manhattan plot of *i*HS statistics calculated by HaploSweep (Zhao et al. 2024). The dashed horizontal line indicates the threshold for outlier *i*HS statistics as specified by the user (in this case *i*HS = 2; statistics above this line are considered outliers). Here, we highlighted the derived C_{-14010} allele associated with lactase persistence in the Bedouin population with a red dot and its corresponding rs identifier (rs145946881). The green and other red dots denote very extreme *i*HS statistics. More explicitly, *i*HS > 4 (red dots) and *i*HS < -4 (green dots) indicate selection on derived alleles (suggestive of a classic selective sweep) and selection on standing variation (suggestive of a soft selective sweep), respectively. **Panel B** shows the decay of EHH (haplotype homozygosity) with increasing distance from the rs145946881 core site, which is associated with lactase persistence. In this EHH line graph, the x-axis shows physical position distance on Chromosome 2 in megabases (Mb), and the y-axis is the probability that two chromosomes are homozygous at all SNPs for the interval from the core site to distance x. Lastly, the blue line represents the decay of homozygosity of chromosomes carrying the ancestral allele at the core, while the red line signifies the decay of homozygosity on chromosomes carrying the derived allele at the core site.

Figure S6: Plots of *i*HS statistics on Chromosome 2, calculated by selscan using unpolarized data, in different populations. **Panels A through C** show Manhattan plots of *i*HS statistics calculated by selscan (Szpiech and Hernandez 2014) using unpolarized data from the Finnish, Bedouin, and Maasai populations, respectively. The dashed horizontal line indicates the threshold for outlier *i*HS statistics as specified by the user (in this case *i*HS = 2; statistics above this line are considered outliers). Here, we highlighted the derived allele

associated with lactase persistence with a red dot and its corresponding rs identifiers in the Finnish (rs4988235), Bedouin (rs41380347), and Massai (rs145946881) populations. The green and other red dots denote very extreme *i*HS statistics. More explicitly, *i*HS > 4 (red dots) and *i*HS < -4 (green dots) indicate selection on derived alleles (suggestive of a classic selective sweep) and selection on standing variation (suggestive of a soft selective sweep), respectively.

Figure S7: Plots of *i*HS statistics on Chromosome 2, calculated by HaploSweep, using the unpolarized data, different populations. **Panels A through C** show Manhattan plots of *i*HS statistics calculated by HaploSweep (Zhao et al. 2024) using unpolarized data from the Finnish, Bedouin, and Maasai populations, respectively. The dashed horizontal line indicates the threshold for outlier *i*HS statistics as specified by the user (in this case *i*HS = 2; statistics above this line are considered outliers). Here, we highlighted the derived allele associated with lactase persistence with a red dot and its corresponding rs identifiers in the Finnish (rs4988235), Bedouin (rs41380347), and Massai (rs145946881) populations. The green and other red dots denote very extreme *i*HS statistics. More explicitly, *i*HS > 4 (red dots) and *i*HS < -4 (green dots) indicate selection on derived alleles (suggestive of a classic selective sweep) and selection on standing variation (suggestive of a soft selective sweep), respectively.

Table S1—Truncated output from selscan and HaploSweep analyses

Population	locusID	chr	Genomic coordinate (GRCh38)	Frequency of derived allele	*i*HS statistic	Method
Finnish	rs4988235	2	135851076	0.590909	3.80086	selscan
Finnish	rs4988235	2	135851076	0.590909	3.68285	HaploSweep
Bedouin	rs41380347	2	135851081	0.271739	3.05771	selscan
Bedouin	rs41380347	2	135851081	0.271739	3.28931	HaploSweep
Maasai	rs145946881	2	135851176	0.546196	4.41326	selscan
Maasai	rs145946881	2	135851176	0.546196	4.49605	HaploSweep

Table S2—Distribution of extreme positive and negative *i*HS statistics (*i*HS < -2 and *i*HS > 2) on Chromosome 2 in the Finnish population from the 1000 Genomes Project calculated by selscan.

	Positive *i*HS	Negative *i*HS	Total
**Polarized dataset**	9,597	10,821	20,418
**Unpolarized dataset**	10,375	13,220	23,595
**Total**	**19,972**	**24,041**	**44,013**

X^2 = 40.5831, df=1, *P* < 0.00001 (two-tailed *P*-value)

Table S3 —Distribution of extreme positive and negative *i*HS statistics (*i*HS < -2 and *i*HS > 2) on Chromosome 2 in the Bedouin population from the HGDP Project calculated by selscan.

	Positive *i*HS	Negative *i*HS	Total
**Polarized dataset**	10,029	11,125	21,154
**Unpolarized dataset**	8,816	14,513	23,329
**Total**	**18,845**	**25,638**	**44,483**

X^2 = 420.06, df=1, *P* < 2.2 x 10^{-16} (two-tailed *P*-value)

Table S4 —Distribution of extreme positive and negative *i*HS statistics (*i*HS < -2 and *i*HS > 2) on Chromosome 2 in the Maasai population from the HapMap Project calculated by selscan.

	Positive *i*HS	Negative *i*HS	Total
**Polarized dataset**	11,609	13,021	24,630
**Unpolarized dataset**	12,554	14,359	26,913
**Total**	**24,163**	**27,380**	**51,543**

X^2 = 1.2052, df=1, *P* =0.2723 (two-tailed *P*-value)

Table S5—Distribution of extreme positive and negative *i*HS statistics (*i*HS < -2 and *i*HS > 2) on Chromosome 2 in the Finnish population from the 1000 Genomes Project calculated by HaploSweep.

	Positive *i*HS	Negative *i*HS	Total
**Polarized dataset**	9,390	10,650	20,040
**Unpolarized dataset**	11,399	11,050	22,449
**Total**	**20,789**	**21,700**	**42,489**

X^2 = 65.1446, df=1, $P < 0.00001$ (two-tailed *P*-value)

Table S6 —Distribution of extreme positive and negative *i*HS statistics (*i*HS < -2 and *i*HS > 2) on Chromosome 2 in the Bedouin population from the HGDP Project calculated by HaploSweep.

	Positive *i*HS	Negative *i*HS	Total
**Polarized dataset**	11,954	10,594	22,548
**Unpolarized dataset**	11,271	14,703	25,974
**Total**	**23,225**	**25,297**	**48,522**

X^2 = 447.46, df = 1, $P < 2.2 \times 10^{-16}$ (two-tailed *P*-value)

Table S7 —Distribution of extreme positive and negative *i*HS statistics (*i*HS < -2 and *i*HS > 2) on Chromosome 2 in the Maasai population from the HapMap Project calculated by HaploSweep.

	Positive *i*HS	Negative *i*HS	Total
**Polarized dataset**	12,582	11,257	23,839
**Unpolarized dataset**	13,729	12,781	26,510
**Total**	**26,311**	**24,038**	**50,349**

X^2 = 4.9017, df = 1, $P = 0.02683$ (two-tailed *P*-value)

Table S8—Distribution of extreme *i*HS statistics and non-extreme *i*HS statistics on Chromosome 2 in the Finnish population from the 1000 Genomes Project calculated by selscan.

	Extreme *i*HS	Non-extreme *i*HS	Total
**Polarized dataset**	20,418	415,072	435,490
**Unpolarized dataset**	23,491	475,203	498,694
**Total**	**43,909**	**890,275**	**934,184**

X^2 = 0.24613, df=1, $P < 0.6190$ (two-tailed *P*-value)

Table S9—Distribution of extreme *i*HS statistics and non-extreme *i*HS statistics on Chromosome 2 in the Bedouin population from the HGDP Panel calculated by selscan.

	Extreme *i*HS	Non-extreme *i*HS	Total
**Polarized dataset**	21,154	437,215	458,369
**Unpolarized dataset**	23,329	499,573	522,902
**Total**	**44,483**	**936,788**	**981,271**

X^2 = 13.282, df=1, $P < 0.0002679$ (two-tailed *P*-value)

Table S10—Distribution of extreme *i*HS statistics and non-extreme *i*HS statistics on Chromosome 2 in the Maasai population from the HapMap Project calculated by selscan.

	Extreme *i*HS	Non-extreme *i*HS	Total
**Polarized dataset**	24,630	514,992	539,622
**Unpolarized dataset**	26,913	568,017	594,930
**Total**	**51,543**	**1,083,009**	**1,134,552**

X^2 = 1.0652, df=1, $P < 0.302$ (two-tailed *P*-value)

Table S11— Distribution of extreme *i*HS statistics and non-extreme *i*HS statistics on Chromosome 2 in the Finnish population from the 1000 Genomes Project calculated by HaploSweep.

	Extreme *i*HS	Non-extreme *i*HS	Total
**Polarized dataset**	20,292	414,956	435,248
**Unpolarized dataset**	23,356	475,087	498,443
**Total**	**43,648**	**890,043**	**933,691**

X^2 = 0.3292, df=1, $P < 0.566153$ (two-tailed *P*-value)

Table S12— Distribution of extreme *i*HS statistics and non-extreme *i*HS statistics on Chromosome 2 in the Bedouin population from the HGDP Panel calculated by HaploSweep.

	Extreme *i*HS	Non-extreme *i*HS	Total
**Polarized dataset**	22,548	436,399	458,947
**Unpolarized dataset**	25,974	496,889	522,863
**Total**	**48,522**	**933,288**	**981,810**

X^2 = 1.543, df=1, $P < 0.2142$ (two-tailed *P*-value)

Table S13—Distribution of extreme *i*HS statistics and non-extreme *i*HS statistics on Chromosome 2 in the Maasai population from the HapMap Project calculated by HaploSweep.

	Extreme *i*HS	Non-extreme *i*HS	Total
**Polarized dataset**	23,839	507,349	531,188
**Unpolarized dataset**	26,510	566,788	593,298
**Total**	**50,349**	**1,074,137**	**1,124,486**

X^2 = 0.24771, df=1, $P < 0.6187$ (two-tailed *P*-value)

Table S14—Proportion of nucleotide sites that are not classified as either ancestral or derived across the autosomal genome.

Chromosome number	Total number of nucleotide sites	Total number of low-confidence nucleotide sites	Proportion of unclassified nucleotide sites
1	244,617,005	42,195,906	0.1724978
2	238,669,784	20,597,194	0.0850444
3	195,672,565	16,398,223	0.0838044
4	187,045,454	14,913,231	0.0797305
5	178,770,047	21,120,058	0.1181409
6	168,120,072	14,518,714	0.0863592
7	156,160,701	21,540,850	0.1379403
8	142,898,027	12,977,994	0.0908200
9	136,184,977	33,447,690	0.2456048
10	131,810,276	14,722,457	0.1116943
11	132,125,409	16,486,958	0.1247826
12	131,156,653	12,413,281	0.0946447
13	112,823,488	25,604,559	0.2269435
14	105,496,039	25,438,373	0.2411311
15	100,550,156	29,038,865	0.2887998
16	88,553,477	25,484,395	0.2877854
17	81,340,382	14,835,543	0.1823884
18	79,248.572	10,682,179	0.1347933
19	55,880,343	14,212,706	0.2543418
20	63,587,922	9,107,578	0.1432281
21	46,070,973	15,090,765	0.3275547
22	49,696,577	21,479,012	0.4322030

Table S15—Summary statistics for unpolarized SNPs on Chromosome 2 in the Finnish population from the 1000 Genomes Project (selscan).

Summary Statistic	Quantitative outcome
Total SNPs with | *i*HS | > 2	23,595
Total SNPs with *i*HS > 2	10,375
Total SNPs with *i*HS < -2	13,220
Count of ancestral alleles (coded as '1') in ALT column	1,049
Count of derived alleles (coded as '0') in REF column	9,276
Proportion of ancestral alleles (coded as '1') in ALT column	0.1011
Proportion of derived alleles (coded as '0') in REF column	0.7017

Table S16—Summary statistics for unpolarized SNPs on Chromosome 2 in the Bedouin population from the HGDP Project (selscan).

Summary Statistic	Quantitative outcome
Total SNPs with | *i*HS | > 2	21,154
Total SNPs with *i*HS > 2	10,029
Total SNPs with *i*HS < -2	11,125
Count of ancestral alleles (coded as '1') in ALT column	4,085
Count of derived alleles (coded as '0') in REF column	2,943
Proportion of ancestral alleles (coded as '1') in ALT column	0.4073
Proportion of derived alleles (coded as '0') in REF column	0.2645

Table S17—Summary statistics for unpolarized SNPs on Chromosome 2 in the Maasai population from the HapMap Project (selscan).

Summary Statistic	Quantitative outcome
Total SNPs with | iHS | > 2	24,638
Total SNPs with *i*HS > 2	11,615
Total SNPs with *i*HS < -2	13,023
Count of ancestral alleles (coded as '1') in ALT column	3,533
Count of derived alleles (coded as '0') in REF column	3,637

Proportion of ancestral alleles (coded as ‘1’) in ALT column	0.3042
Proportion of derived alleles (coded as ‘0’) in REF column	0.2793

Table S18—Summary statistics for unpolarized SNPs on Chromosome 2 in the Finnish population from the 1000 Genomes Project (HaploSweep).

**Summary Statistic**	**Quantitative outcome**
Total SNPs with | *i*HS | > 2	22,449
Total SNPs with *i*HS > 2	11,399
Total SNPs with *i*HS < -2	11,050
Count of ancestral alleles (coded as ‘1’) in ALT column	2,677
Count of derived alleles (coded as ‘0’) in REF column	6,493
Proportion of ancestral alleles (coded as ‘1’) in ALT column	0.2348
Proportion of derived alleles (coded as ‘0’) in REF column	0.5876

Table S19—Summary statistics for unpolarized SNPs on Chromosome 2 in the Bedouin population from the HDGP Project (HaploSweep).

**Summary Statistic**	**Quantitative outcome**
Total SNPs with | *i*HS | > 2	22,548
Total SNPs with *i*HS > 2	11,954
Total SNPs with *i*HS < -2	10,594
Count of ancestral alleles (coded as ‘1’) in ALT column	3,774
Count of derived alleles (coded as ‘0’) in REF column	3,397
Proportion of ancestral alleles (coded as ‘1’) in ALT column	0.3157
Proportion of derived alleles (coded as ‘0’) in REF column	0.3207

Table S20—Summary statistics for unpolarized SNPs on Chromosome 2 in the Maasai population from the HapMap Project (HaploSweep).

Summary Statistic	Quantitative outcome
Total SNPs with | *i*HS | > 2	23,846
Total SNPs with *i*HS > 2	12,588
Total SNPs with *i*HS < -2	11,258
Count of ancestral alleles (coded as ‘1’) in ALT column	3,465
Count of derived alleles (coded as ‘0’) in REF column	3,441
Proportion of ancestral alleles (coded as ‘1’) in ALT column	0.2753
Proportion of derived alleles (coded as ‘0’) in REF column	0.3056

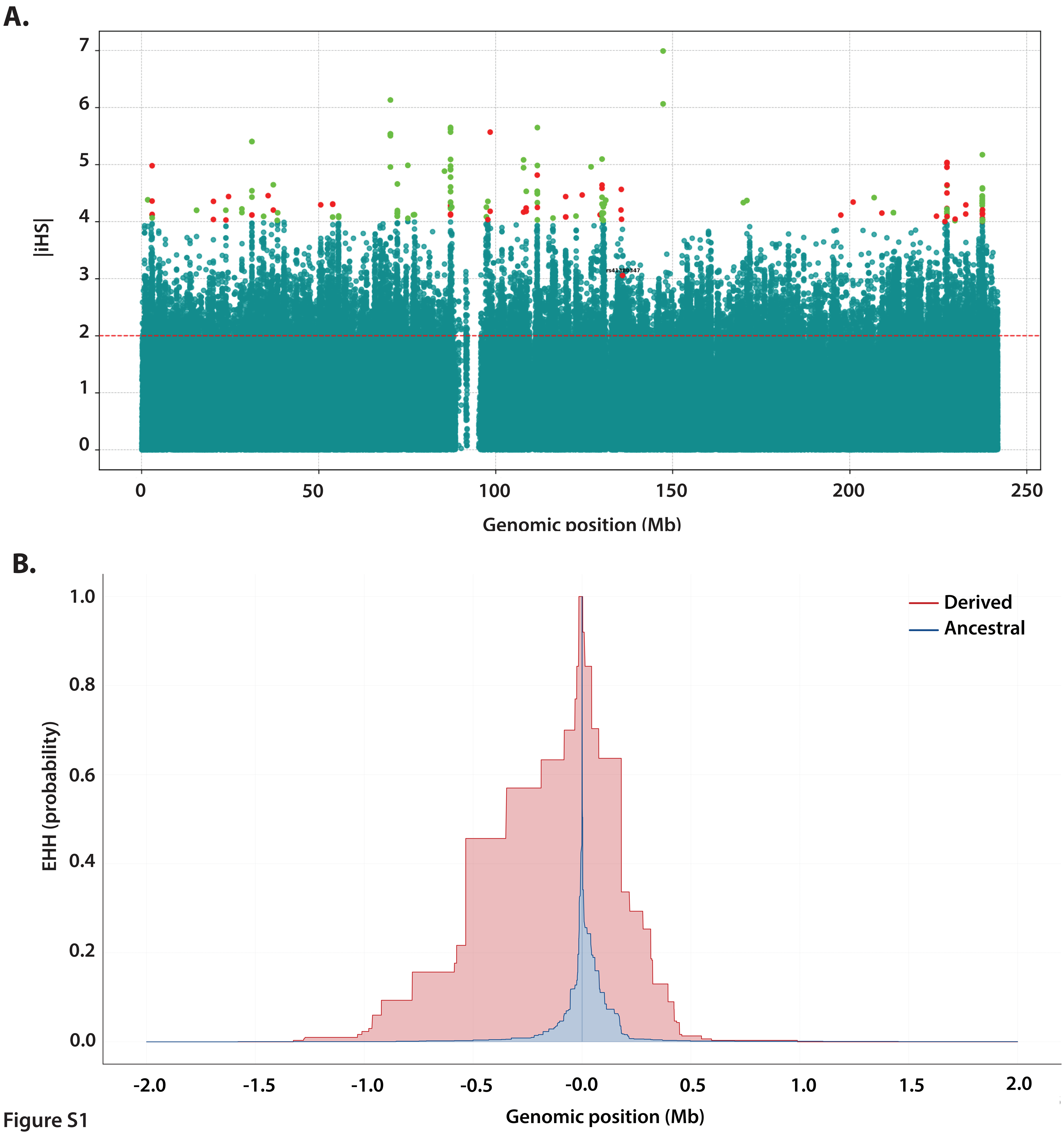
A.
|iHS|
Genomic position (Mb)
B.
EHH (probability)
Genomic position (Mb)
Derived
Ancestral

Figure S1

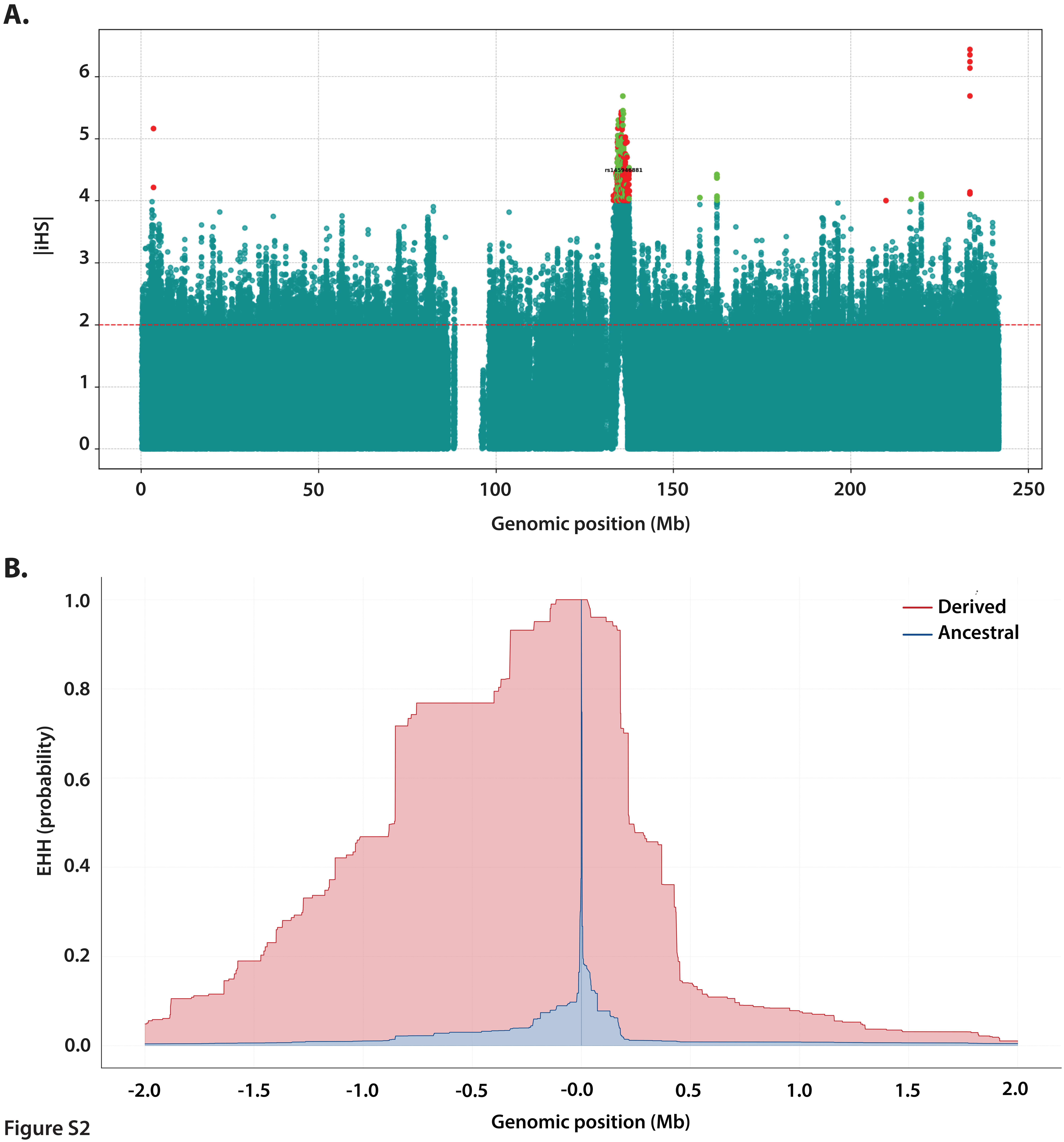
A.
|iHS|
6
5
4
3
2
1
0
rs145946881
0
50
100
150
200
250
Genomic position (Mb)
B.
EHH (probability)
1.0
0.8
0.6
0.4
0.2
0.0
Derived
Ancestral
-2.0
-1.5
-1.0
-0.5
-0.0
0.5
1.0
1.5
2.0
Genomic position (Mb)

Figure S2

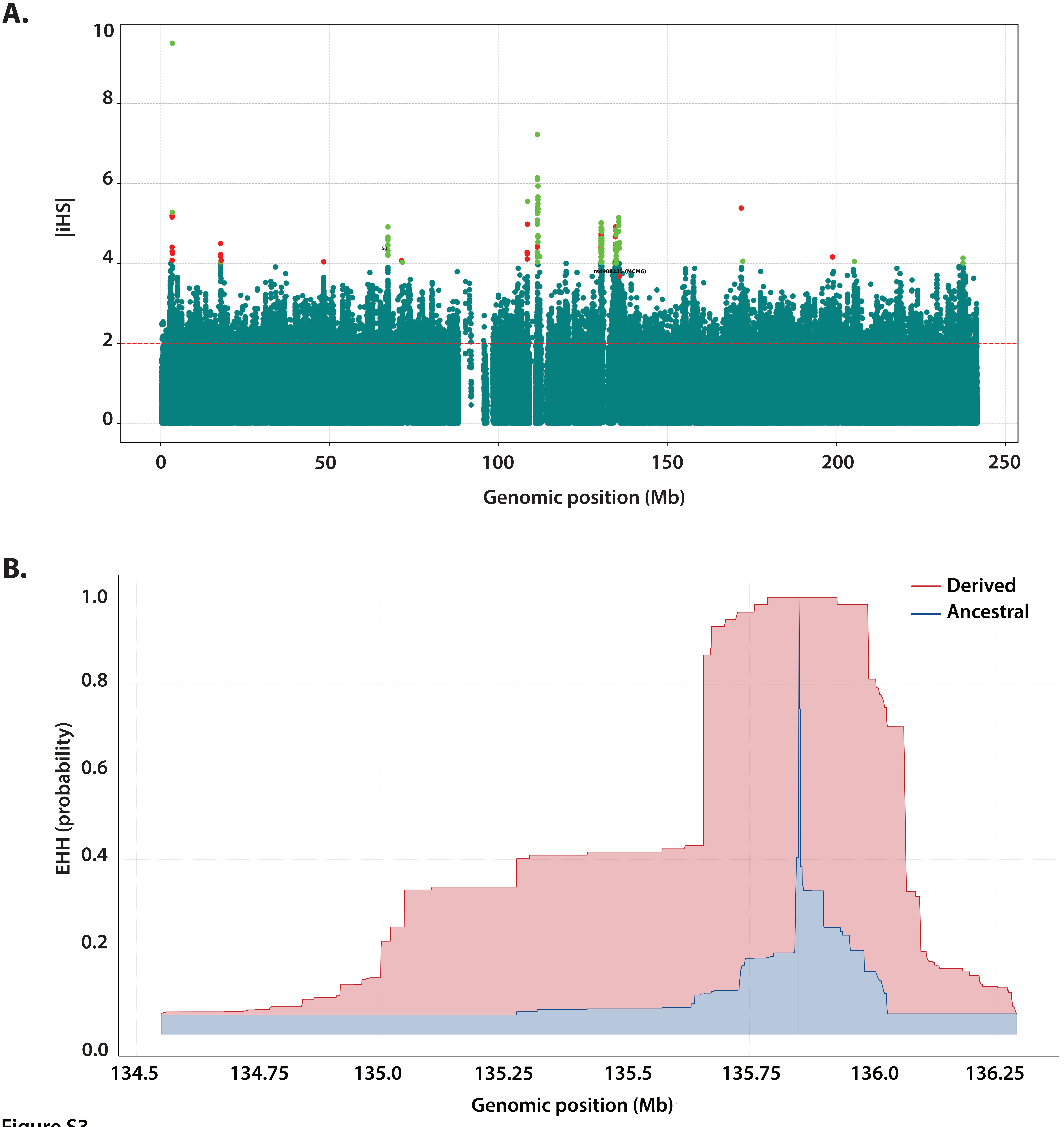
A.
10
8
6
4
2
0
|iHS|
rs4988235 (MCM6)
0
50
100
150
200
250
Genomic position (Mb)
B.
Derived
Ancestral
1.0
0.8
0.6
0.4
0.2
0.0
EHH (probability)
134.5
134.75
135.0
135.25
135.5
135.75
136.0
136.25
Genomic position (Mb)

Figure S3

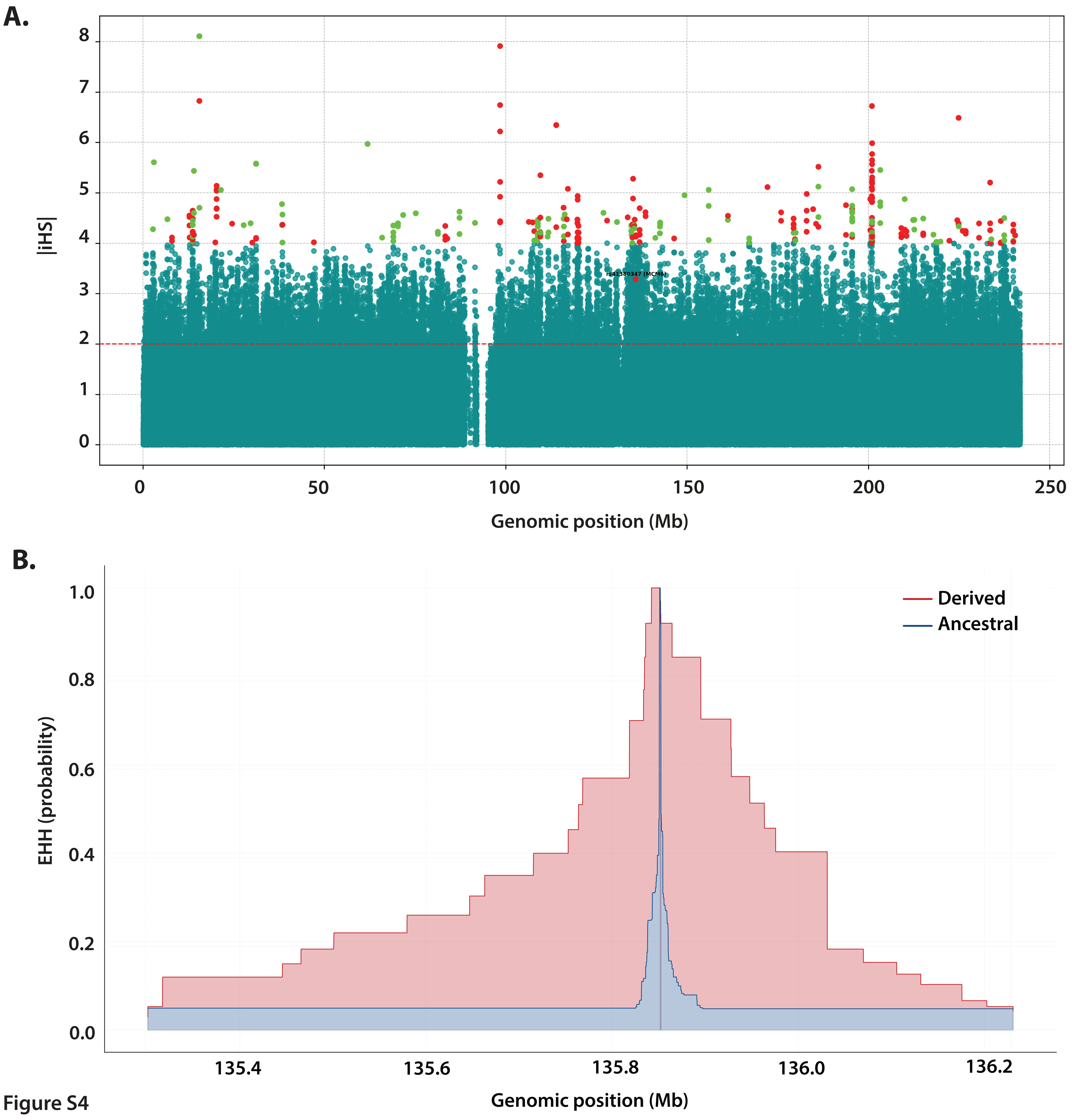
A.
8
7
6
5
4
3
2
1
0
|iHS|
rs41380347 (MCM6)
0
50
100
150
200
250
Genomic position (Mb)
B.
1.0
0.8
0.6
0.4
0.2
0.0
EHH (probability)
Derived
Ancestral
135.4
135.6
135.8
136.0
136.2
Genomic position (Mb)

Figure S4

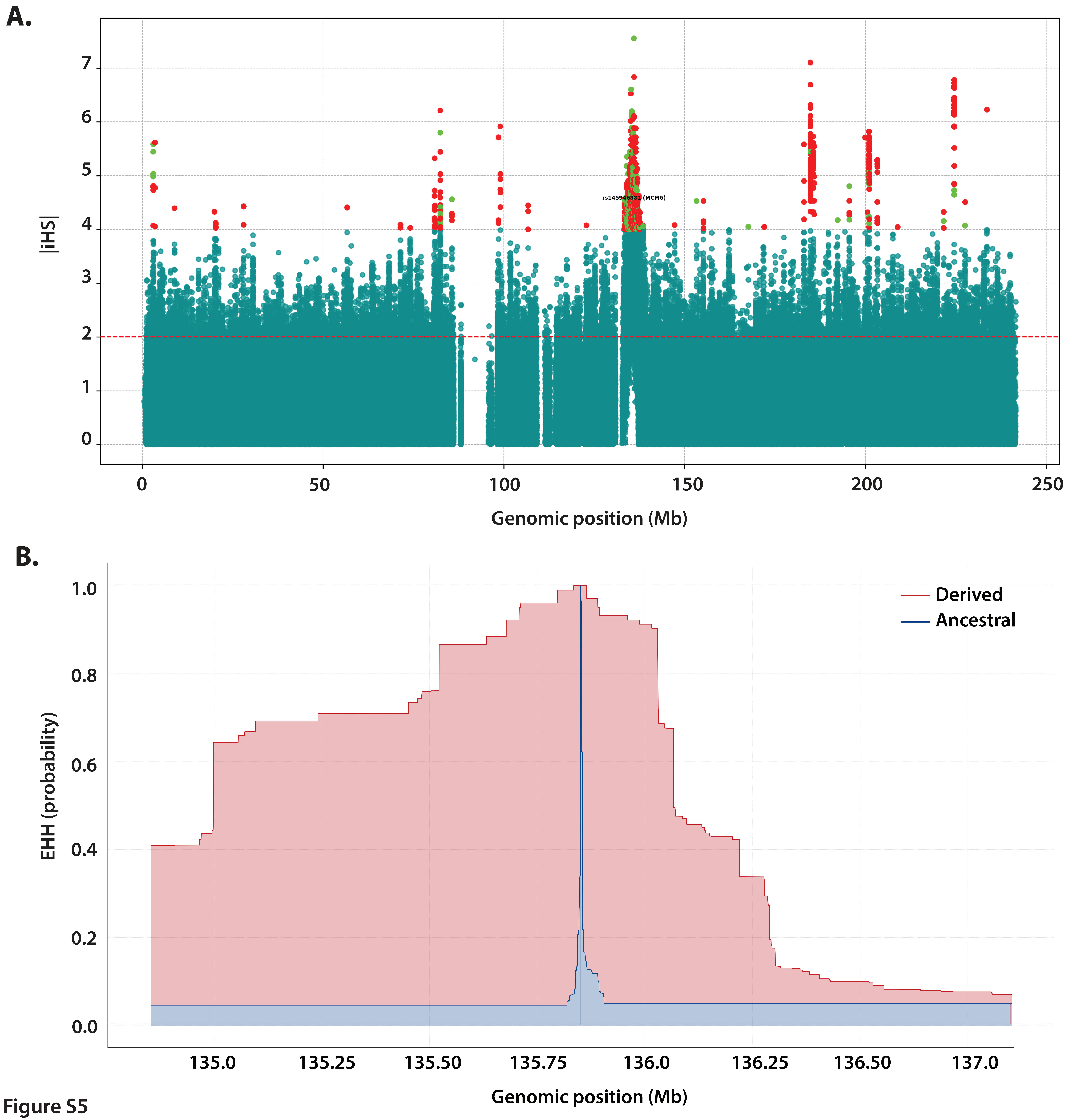
A.
|iHS|
7
6
5
4
3
2
1
0
rs145946881 (MCM6)
0
50
100
150
200
250
Genomic position (Mb)
B.
EHH (probability)
1.0
0.8
0.6
0.4
0.2
0.0
Derived
Ancestral
135.0
135.25
135.50
135.75
136.0
136.25
136.50
137.0
Genomic position (Mb)

Figure S5

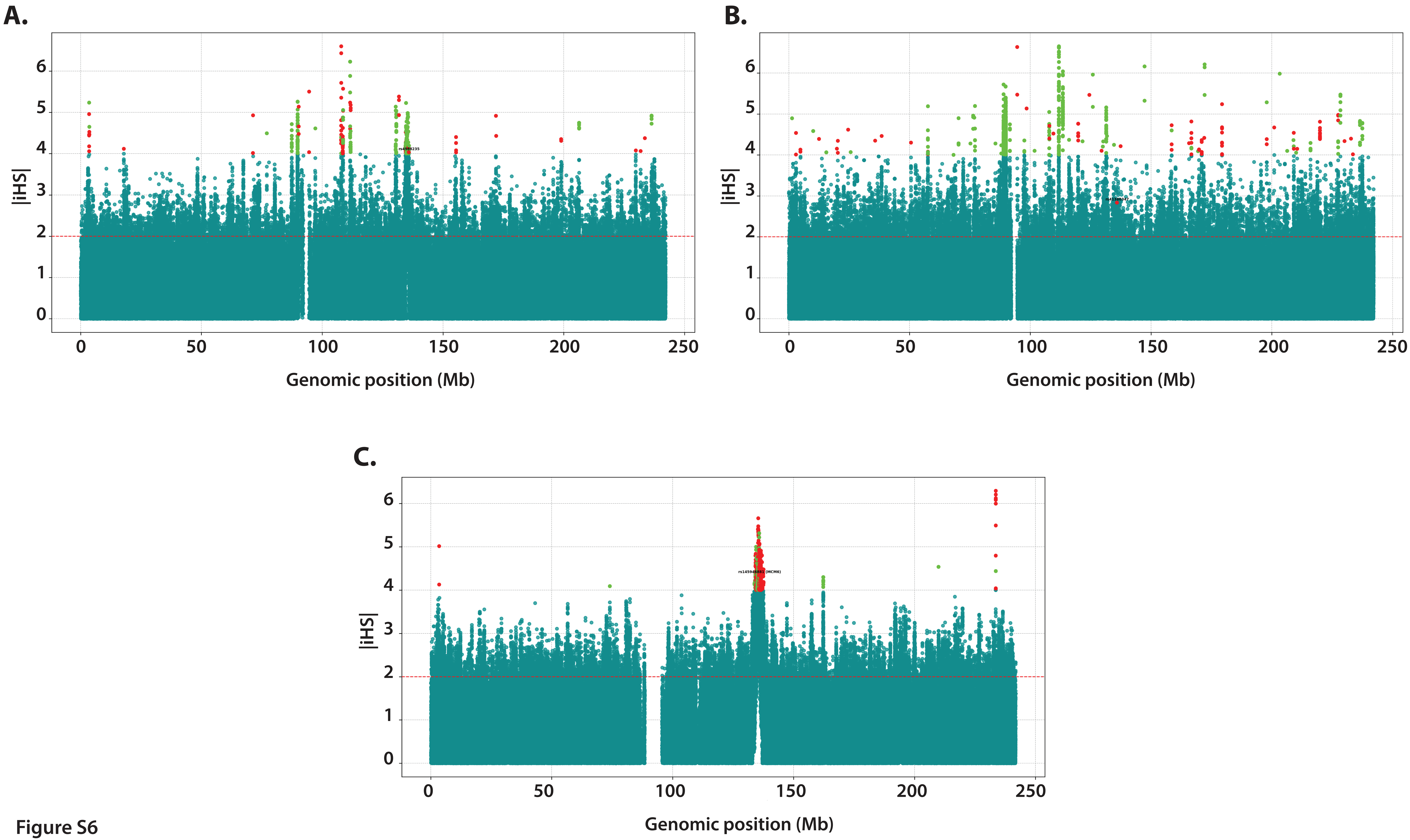
A.
6
5
4
3
2
1
0
|iHS|
rs4988235
0
50
100
150
200
250
Genomic position (Mb)
B.
6
5
4
3
2
1
0
|iHS|
rs41380347
0
50
100
150
200
250
Genomic position (Mb)
C.
6
5
4
3
2
1
0
|iHS|
rs145946881 (MCM6)
0
50
100
150
200
250
Genomic position (Mb)

Figure S6

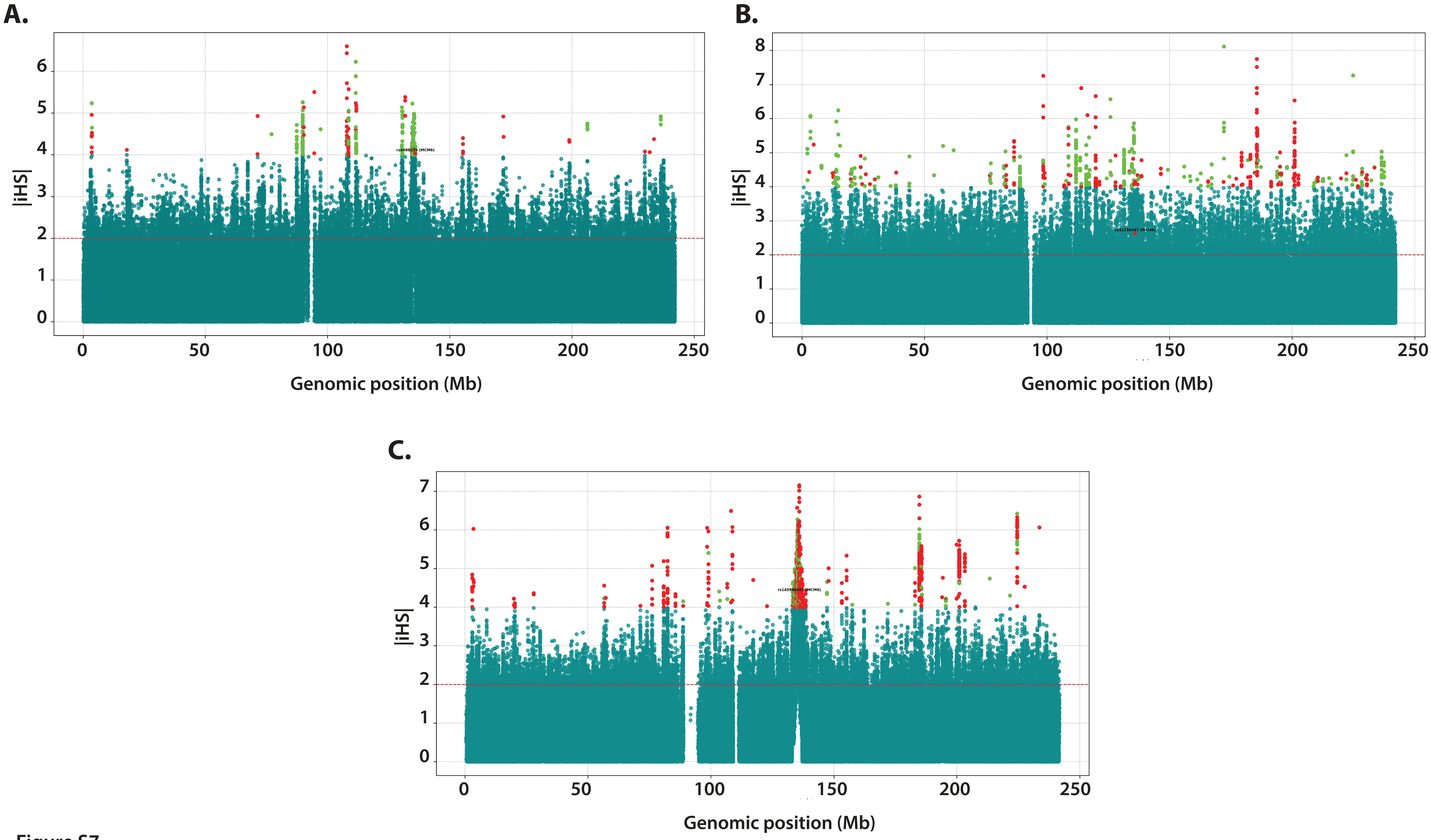

Figure S7
